# Daflon and Centrum mitigate vancomycin-induced nephrotoxicity in rats by ameliorating oxidative stress, DNA damage, apoptosis, and inflammation

**DOI:** 10.3389/ftox.2025.1673083

**Published:** 2025-09-15

**Authors:** Hanem F. El-Gendy, Soaad Salamah, Eman Elhusseiny, Hazim O. Khalifa, Hossny A. El-Banna, Taha A. Attia, Shaimaa Selim, Saber El Hanbally

**Affiliations:** ^1^ Department of Pharmacology, Faculty of Veterinary Medicine, University of Sadat City, Sadat City, Egypt; ^2^ Department of Veterinary Medicine, College of Food and Agriculture, United Arab Emirates University, Al Ain, United Arab Emirates; ^3^ United Arab Emirates University (UAEU) Center for Public Policy and Leadership, United Arab Emirates University, Al Ain, United Arab Emirates; ^4^ Department of Pharmacology, Faculty of Veterinary Medicine, Cairo University, Giza, Egypt; ^5^ Department of Nutrition and Clinical Nutrition, Faculty of Veterinary Medicine, Menoufia University, Shibin El-Kom, Egypt

**Keywords:** Daflon, Centrum, vancomycin, antioxidant, inflammatory cytokine, DNA damage, nephrotoxicity, oxidative stress

## Abstract

**Background:**

Vancomycin (VM) is widely used for treating life-threatening infections caused by Gram-positive bacteria resistant to other antibiotics. However, its nephrotoxic effects limit clinical use.

**Objective:**

This study aimed to evaluate the protective effects of Daflon (DF) and Centrum (CE) against VM-induced nephrotoxicity in male rats.

**Methods:**

Fifty healthy male Wistar rats were randomly divided into five groups. Group 1 (negative control) received saline intraperitoneally (IP) for 7 days followed by oral distilled water for 7 days. Group 2 (positive control) received VM (400 mg/kg BW, IP) for 7 days. Group 3 received VM for 7 days followed by DF (100 mg/kg BW, oral) for 7 days. Group 4 received VM for 7 days followed by CE (15 mg/kg BW, oral) for 7 days. Group 5 received VM for 7 days followed by combined DF and CE treatment for 7 days. Blood and kidney samples were collected for hematological, biochemical, molecular, comet assay, and histopathological evaluations.

**Results:**

VM administration significantly elevated serum creatinine, urea, and uric acid levels (p < 0.01), increased renal malondialdehyde (MDA), and reduced catalase (CAT) and superoxide dismutase (SOD) activities (p < 0.05). It also induced marked histological changes and increased DNA fragmentation. DF and CE, particularly in combination (Group 5), significantly reduced renal injury, DNA fragmentation, and histopathological alterations. The protective effect followed the order: G5 > G4 > G3 > G2. Furthermore, VM upregulated *PARP1, RIP1, KIM1, TNF-α, and IL-1β* expression, which were markedly downregulated by DF and CE.

**Conclusion:**

DF and CE attenuated VM-induced nephrotoxicity through antioxidant, anti-inflammatory, and DNA-protective mechanisms. Their combination provided superior renal protection by reducing oxidative stress, inflammation, and apoptosis, while enhancing antioxidant defenses and DNA repair capacity.

## Graphical Abstract

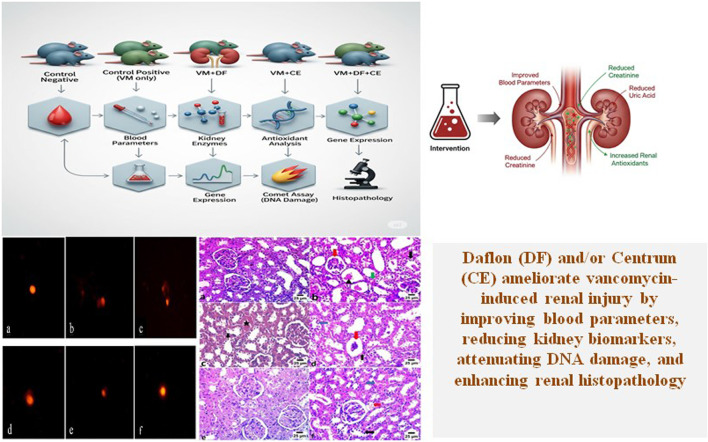

## 1 Introduction

Acute kidney disease (AKD) is a serious medical condition characterized by a rapid and reversible decline in renal function. It is associated with a high risk of permanent kidney damage, poor prognosis, and substantial healthcare costs. Several essential medications have been reported to induce AKD, thereby limiting their clinical utility (Abd et al., 2023). Vancomycin (VM), a glycopeptide antibiotic, belongs to a class that includes teicoplanin, decaplanin, and ramoplanin—cyclic, glycosylated polycyclic peptides. Due to their toxicity, these drugs are typically reserved for patients with β-lactam allergies (Van Groesen et al., 2022). VM is commonly used to treat infections caused by aerobic and anaerobic Gram-positive bacteria, including *Staphylococcus epidermidis* and methicillin-resistant *Staphylococcus aureus* (MRSA) (Li et al., 2022). It is also prescribed for various infections such as *Clostridioides difficile*, endocarditis, septicemia, meningitis, peritonitis, joint, and skin infections (Patel et al., 2024). VM exerts its antimicrobial effect by inhibiting bacterial cell wall synthesis via binding to the D-Ala-D-Ala terminal of peptidoglycan precursors (Wang et al., 2018). However, nephrotoxicity remains a significant adverse effect (Alosaimy et al., 2024), and its underlying mechanisms are not fully understood (Kucukler et al., 2020). Studies suggest that oxidative stress, inflammation, and apoptosis are central to VM-induced nephrotoxicity, particularly affecting renal proximal tubular epithelial cells and leading to tubular injury and ischemia (Kan et al., 2022). Free radicals generated during VM treatment can disrupt endogenous antioxidant defenses, such as superoxide dismutase (SOD), contributing to renal impairment (Yu et al., 2022). Additionally, VM can induce DNA damage, mitochondrial dysfunction, and activation of apoptosis-related pathways, including cytochrome c release and caspase activation (Humanes et al., 2015). VM has also been shown to overactivate poly (ADP-ribose) polymerase-1 (PARP-1), further promoting apoptosis (Dalaklioglu et al., 2010). Therefore, protecting renal tissue from oxidative and DNA damage may enhance the therapeutic benefits of VM.

Natural compounds with antioxidant properties offer potential protection against VM-induced renal injury. Daflon (DF) is an oral micronized flavonoid formulation containing 90% diosmin and 10% hesperidin. Flavonoids exhibit various biological activities, including antioxidant effects (Abd El-Hafeez et al., 2018; Abd et al., 2023). They reduce oxidative stress by neutralizing superoxide radicals, which results in the formation of hydrogen peroxide and anti-inflammatory flavonoid radicals (Abd et al., 2023). Centrum (CE) is a multivitamin and multimineral supplement containing essential vitamins (A, B1, B2, B6, B12, C, D3, E, K) and minerals (calcium, magnesium, iron, zinc, selenium, phosphorus, and others), formulated to support nutritional balance and physiological wellness (Alahmar and Singh, 2022). In AKD, deficiencies in vitamins such as B1, B2, B6, C, D, and K are common (Juszczak et al., 2023; Li et al., 2024). These vitamins and minerals exhibit antioxidant, anti-inflammatory, and anti-apoptotic properties. For example, vitamin C mitigates organ damage by reducing oxidative stress; vitamin E prevents lipid peroxidation (Soltani et al., 2020); and vitamin D3 enhances antioxidant defenses by upregulating enzymes like SOD (Al-Sroji et al., 2023). Vitamin B2 has been shown to increase renal antioxidant enzymes (GR, GSH, SOD, CAT) in toxicity models (Alhazza et al., 2020). Lutein, another CE component, reduces inflammation by inhibiting NF-κB and PI3K signaling pathways (Gan and Azrina, 2016). Moreover, phosphorus supports antioxidant function, and its deficiency has been linked to increased ROS and lipid peroxidation (Kayoumu et al., 2023). Magnesium deficiency impairs mitochondrial function and promotes ROS generation (Liu and Dudley, 2020).

Based on this evidence, we hypothesized that DF and CE may offer protective effects against VM-induced nephrotoxicity by modulating oxidative stress, inflammation, and apoptotic pathways. Therefore, the present study was designed to investigate the potential therapeutic benefits of DF, CE, or their combination in mitigating VM-induced acute kidney injury and DNA damage in rats, and to explore their mechanisms of action through evaluation of biochemical, inflammatory, and apoptotic markers.

## 2 Materials and methods

### 2.1 Studied materials

Vancomycin was obtained as a 500 mg powder for injection (Vancobact^®^ One, Egypharma, Nasr City, Cairo, Egypt). Daflon was purchased as Daflon^®^ 1,000 mg tablets, each containing a purified micronized flavonoid fraction composed of 900 mg diosmin (90%) and 100 mg flavonoids expressed as hesperidin (10%) (Serdia Pharmaceuticals Private Limited, India). Centrum was acquired as Centrum with Lutein^®^, which contains a blend of vitamins, minerals, and lutein, as detailed in Table 1. Both DF and CE were sourced from Serdia Pharmaceuticals Private Limited, India.

**TABLE 1:** Active ingredients of Centrum.

Vitamins	Weights	Vitamins	Weights	Minerals	Weights	Minerals	Weights
Vit. A	400 µg	Vit. B6	2 mg	Calcium	162 µg	Potassium	40 mg
Vit. E	14.9 IU	Vit. B12	1 µg	Iodine	100 µg	Chromium	40 µg
Vit. C	60 mg	Vit. D3	200 IU	Iron	10 mg	Molybdenum	50 µg
Folic acid	195 µg	Biotin	100 µg	Magnesium	50 mg	Selenium	30 µg
Vit. B1	1.4 mg	Pantothenic acid	6 mg	Copper	0.5 mg	Zinc	5 mg
Vit. B2	1.6 mg	Vit. K1	30 µg	Manganese	1 mg	Other ingredients	
Niacinamide	18 mg			Phosphorus	125 mg	Lutein	250 µg

### 2.2 Rats and trial design

Fifty healthy male albino Wistar rats, weighing between 120 and 140 g, were obtained from the Laboratory Animal Farm in Giza, Egypt. The animals were housed in the animal facility of the Faculty of Veterinary Medicine, University of Sadat City, under controlled environmental conditions: 23 °C ± 2 °C temperature, 50% relative humidity, and a 12-h light/dark cycle. Rats had free access to water and a standard pellet diet. A 2-week acclimatization period was allowed prior to the start of the experiment.

At the beginning of the study, rats were weighed and randomly allocated into five groups of ten animals each (Table 2). All experimental protocols were approved by the Ethical Committee of the Faculty of Veterinary Medicine, University of Sadat City, Egypt, in accordance with institutional and national guidelines for the care and use of laboratory animals (Approval No. VUSC-044-1-20). The experimental design followed the protocol of He et al. (2021) to evaluate the protective effects of Daflon (DF) and Centrum (CE) against VM-induced nephrotoxicity. The study lasted 14 days.

**TABLE 2:** Experimental design of the study.

Experimental period		
Treatments	From the 1st to the 7th day of the experiment	From the 8th to the 14th day of the trial
Control	Normal saline IP 1 mL	Distilled water orally 1 mL
VM	VM (IP) (400 mg/kg BW)	Scarify rats after first 7 days
VM + DF	VM (IP) (400 mg/kg BW)	DF (orally) (100 mg/kg BW)
VM + CE	VM (IP) (400 mg/kg BW)	CE (orally) (15 mg/kg BW)
VM + DF + CE	VM (IP) (400 mg/kg BW)	DF (orally) (100 mg/kg BW) + CE (orally) (15 mg/kg BW)

Group 1 (negative control) received 1 mL of normal saline intraperitoneally (IP) for 7 days, followed by 1 mL of distilled water orally for the next 7 days. Group 2 (positive control) received VM (400 mg/kg body weight) via IP injection for 7 days (El Latif et al., 2023). Group 3 received VM (400 mg/kg BW, IP) for 7 days, followed by oral administration of DF (100 mg/kg BW) once daily for another 7 days (Abd et al., 2023). Group 4 received VM (400 mg/kg BW, IP) for 7 days, followed by oral CE (15 mg/kg BW) once daily for 7 days (Paget and Barnes 1964). Group 5 was treated with VM (400 mg/kg BW, IP) for 7 days, followed by a combination of DF (100 mg/kg BW, oral) and CE (15 mg/kg BW, oral) once daily for 7 days.

### 2.3 Sampling

At the end of the experimental period (on day 7 for Group 2 and day 14 for Groups 1, 3, 4, and 5), the rats were subjected to a 12-h fasting period prior to sample collection. Under diethyl ether anesthesia (Sigma Chemical Co., St. Louis, MO, United States), blood samples were collected from six rats per group via retro-orbital puncture. Each blood sample was divided into two portions: the first was placed in EDTA-containing tubes for hematological analysis, while the second was transferred into non-heparinized centrifuge tubes, allowed to clot, and then centrifuged at 3,000 rpm for 15 min to separate the serum. The collected serum was stored at −20 °C for subsequent biochemical assays.

After blood collection, the rats were euthanized by cervical dislocation to obtain kidney tissue samples. The kidneys were immediately excised, rinsed with cold saline to remove residual blood, and blotted dry with filter paper. Each kidney was then divided into two portions. One portion was stored at −80 °C for gene expression analysis, comet assay, and other biochemical investigations. The other portion was fixed in 10% neutral buffered formalin for histopathological examination.

### 2.4 Growth performance

Initial body weight (BW), BW after 1 week, and final BW were recorded to assess body weight changes in the rats. Feed intake (FI) was calculated by subtracting the remaining feed from the amount provided the previous day. Weight gain (WG) was measured over the 2-week experimental period. The feed conversion ratio (FCR) was calculated by dividing WG by FI, as described by Abd et al. (2023) and Achi et al. (2023).

### 2.5 Absolute and relative body and organ weights

On the day of sacrifice, prior to euthanasia, the experimental rats were weighed. After sacrifice, the kidneys were carefully excised, cleared of surrounding tissues, and weighed. The relative organ weight (ROW) of the kidneys was calculated using the following formula (Zheng et al., 2024):

$$\text{ROW}=\left[\text{Kidney weight }\left(g\right)\;/\text{ Body weight of rat }\left(g\right)\right]\times 100$$

### 2.6 Hematological investigation

After collection, blood samples were immediately analyzed using an automated hematology analyzer and cell counter (Sysmex F-800, Tokyo, Japan) to assess the following hematological parameters: hemoglobin (Hb) concentration, packed cell volume (PCV %), total leukocyte count (TLC), platelet count (Plt), red blood cell (RBC) count, and differential leukocyte counts.

### 2.7 Biochemical evaluation

Serum levels of albumin, uric acid, total protein, creatinine, and urea were measured using commercial kits following the manufacturer’s instructions (Bio-Diagnostic, Dokki, Giza, Egypt).

### 2.8 Preparation of renal tissue homogenate

Renal tissues were weighed and prepared for analysis by adding ice-cold phosphate-buffered saline (pH 7.4) at a volume corresponding to 25% w/v based on tissue weight. Samples were placed on ice and homogenized using an ultrasonic sonicator (Qsonica, Newtown, CT, United States) to disrupt cell walls and release intracellular contents. The homogenates were then centrifuged at 1700 rpm for 10 min in a refrigerated centrifuge. The resulting supernatants were collected into fresh Eppendorf tubes and stored at −80 °C for subsequent biochemical analysis of oxidative stress and antioxidant biomarkers (Caio-Silva et al., 2020).

### 2.9 Oxidant/antioxidant biomarkers in tissue homogenate

Antioxidant enzyme activities and oxidative stress biomarkers were measured using commercially available colorimetric assay kits (Biodiagnostic, Dokki, Giza, Egypt), following the manufacturer’s instructions. Renal malondialdehyde (MDA) levels were assessed as an indicator of lipid peroxidation (Kartavenka et al., 2020), while superoxide dismutase (SOD) activity (Misra and Fridovich, 1972) and catalase activity (Góth, 1991) were also determined.

### 2.10 Kidney injury marker genes (quantitative RT-PCR)

Total RNA was extracted from tissue samples using the QIAamp RNeasy Mini Kit (Qiagen, Germany). Briefly, 100 mg of tissue was homogenized in 600 µL of RLT buffer containing 10 µL of β-mercaptoethanol per milliliter. Homogenization was performed using the Qiagen TissueLyser with adaptor sets and clamps, operating at 30 Hz for 2 min. The resulting lysate was mixed with an equal volume of 70% ethanol. RNA purification was then completed following the manufacturer’s protocol for total RNA extraction from animal tissue (Qiagen, Germany).

Reverse transcription and real-time PCR (RT-qPCR) were carried out using specific oligonucleotide primers (listed in Table 3, synthesized by OligoTM). Each 20 µL reaction mixture included 3 µL of RNA template, 4 µL of nuclease-free water, 1 µL of RT enzyme mix (20X), 10 µL of HERA SYBR^®^ Green RT-qPCR Master Mix (2X), and 1 µL of each primer (200 nM). Reactions were run on a real-time PCR system (Applied Biosystems). Amplification curves and cycle threshold (Ct) values were obtained, and relative gene expression levels were analyzed using the ΔΔCt method, comparing each sample to the positive control group (Livak and Schmittgen, 2001).

**TABLE 3:** Primers sequences, amplicon sizes, target genes, and cycling conditions for SYBR green RT-PCR.

Target gene	Primers sequences	References
KIM-1	GGT​CAC​CCT​GTC​ACA​ATT​CC	Mohamed et al. (2021)
	CTC​GGC​AAC​AAT​ACA​GAC​CA	
PARP-1	GAC​ACG​GTT​TTC​ATT​GTC​CA	El Agaty (2018)
	TGA​GAC​TCA​GCA​GGC​AGG​TA	
RIP-1	AGG​TAC​AGG​AGT​TTG​GTA​TGG​GC	Cui et al. (2016)
	GGT​GGT​GCC​AAG​GAG​ATG​TAT​G	
IL-1β	CAC​CTC​TCA​AGC​AGA​GCA​CAG	Alkhedaide et al. (2016)
	GGG​TTC​CAT​GGT​GAA​GTC​AAC	
TNF-α	AAA​TGG​GCT​CCC​TCT​CAT​CAG​TTC	
	TCT​GCT​TGG​TGG​TTT​GCT​ACG​AC	
β-actin	AAG​TCC​CTC​ACC​CTC​CCA​AAA​G	
	AAG​CAA​TGC​TGT​CAC​CTT​CCC	

The RT-PCR was performed under the following cycling conditions: reverse transcription at 55 °C for 15 min, enzyme activation at 95 °C for 5 min, followed by 40 cycles of denaturation at 95 °C for 10 s, annealing (or strengthening) at 58 °C–60 °C for 10 s depending on the target gene, and extension at 60 °C for 30 s. Primers for the target genes *KIM-1, PARP-1, RIP-1, IL-1β, TNF-α*, and *β-actin* were used as listed in Table 3.

### 2.11 Comet assay for DNA damage (single cell gel electrophoresis)

To assess potential DNA damage resulting from the various treatments, the Comet assay (single-cell gel electrophoresis, SCGE) was performed according to the method described by Singh et al. (1988). The comet assay was performed using the Comet Assay Kit (Trevigen, Gaithersburg, MD, United States; Cat. No. 4250-050-K) according to the manufacturer’s instructions. Slides were examined using a fluorescence microscope (Olympus BX53, Tokyo, Japan) equipped with a ×40 objective lens and a FITC filter set, and images were captured with a DP74 digital camera and analyzed using CometScore 2.0 software (TriTek Corp., Sumerduck, VA, United States). The assay procedure followed the method of Singh et al. (1988) with minor modifications.

Kidney tissue samples stored at −80 °C were used for the comet assay. Single-cell suspensions were prepared as follows: A small tissue block (∼25–40 mg) was kept on dry ice and finely diced with a sterile, chilled scalpel. The tissue was gently homogenized in 1 mL ice-cold homogenization buffer (PBS with 20 mM EDTA and 10% DMSO) using a glass Dounce tissue grinder (∼10 × passes on ice). The homogenate was passed through a 70 µm nylon cell strainer into a pre-chilled tube to remove debris. Cells were pelleted at 200 × g for 5 min at 4 °C, resuspended gently in 300 µL ice-cold PBS, and kept on ice until embedding in low-melting-point agarose on microscope slides. Finally, slides were processed under alkaline conditions (pH > 13), electrophoresed at 1 V/cm for 20 min, neutralized, stained (e.g., ethidium bromide), and analyzed using fluorescence microscopy. At least 100 nuclei per sample were scored for % tail DNA using automated image analysis software (Jackson et al., 2013).

This technique detects DNA strand breaks and alkali-labile sites by evaluating the migration of DNA fragments under an electric field. Cells from the control and treated groups were embedded in an agarose gel layer on microscope slides. The slides were then immersed in a lysis solution to dissolve cell membranes and proteins, leaving the nuclear DNA immobilized within the gel. After lysis, the slides were incubated in an alkaline buffer to denature the DNA, allowing strand breaks to unwind the supercoiled DNA and relax the structure. An electric field was then applied, causing fragmented DNA to migrate from the nucleus toward the anode, forming a “comet tail.” The extent of DNA migration reflects the degree of damage: the more relaxed and fragmented the DNA, the longer the tail. Following electrophoresis, the slides were neutralized, fixed in ethanol, and stained with GelRed^®^, a fluorescent dye that binds specifically to DNA. The slides were examined under a fluorescence microscope, and image analysis software was used to measure parameters such as tail length and the percentage of DNA in the tail. The tail moment, calculated by multiplying tail length by tail DNA percentage, served as a quantitative indicator of DNA damage. DNA damage was classified into five grades based on tail length: Grade 0 (<5%) indicating no damage; Grade I (5%–20%) slight damage; Grade II (20%–40%) moderate damage; Grade III (40%–95%) severe damage; and Grade IV (>95%) indicating extensive DNA damage.

### 2.12 Histopathological investigation

Kidney tissue samples were fixed in 10% neutral buffered formalin, washed with water, dehydrated through a graded series of ethanol, cleared in xylene, and embedded in paraffin. Thin sections (4–6 µm) were then prepared, stained with hematoxylin and eosin (H&E), and examined as described by Bancroft (2008). Validation of the scoring system’s repeatability and biological relevance, and employing appropriate statistical analysis to produce reliable results in comparative and toxicological pathology studies (Toprak et al., 2020).

### 2.13 Statistical analysis

Data were expressed as mean ± standard error of the mean (SEM). One-way ANOVA was performed to compare differences among groups, followed by Duncan’s Multiple Range Test for post-hoc analysis using SPSS version 21. Statistical significance was considered at P ≤ 0.05.

## 3 Results

### 3.1 Growth findings, absolute and relative kidney weights

In the current study, the VM + DF + CE group showed a significant improvement in body weight (BW) and weight gain (WG), along with a notable reduction in feed conversion ratio (FCR) compared to the other VM-treated groups (p < 0.05) (Table 4). No significant differences were observed in relative kidney weights among the treatment groups (Table 5). In the VM group, the initial BW was 122.00 ± 2.54 g, increasing to 139.00 ± 2.44 g after 1 week. The feed intake (FI) was 55.00 ± 2.73 g, resulting in a WG of 17.00 ± 2.54 g and an FCR of 3.44 ± 0.16.

**TABLE 4:** Effect of DF and CE with VM on body weight parameters.

Treatments						
Items	Control	VM	VM + DF	VM + CE	VM + DF + CE	P-Value
Initial BW, g	123.00 ± 2.54	122.00 ± 2.54	122.00 ± 2.54	123.00 ± 2.54	123.00 ± 2.54	0.901
Final BW, g	176.00 ± 5.78^a^	139.00 ± 2.44^c^	150.00 ± 4.74^b^	151.00 ± 5.37^b^	166.00 ± 2.44^a^	<0.001
FI, g	160.00 ± 6.80^a^	55.00 ± 2.73^d^	111.00 ± 6.78^c^	122.00 ± 8.74^bc^	133.00 ± 6.81^b^	<0.001
WG, g	49.00 ± 3.67^a^	17.00 ± 2.54^c^	28.00 ± 2.54^b^	28.00 ± 4.06^b^	43.00 ± 4.06^a^	<0.001
FCR (%)	3.30 ± 0.13^b^	3.44 ± 0.16^b^	3.99 ± 0.024^a^	4.58 ± 0.05^a^	3.90 ± 3.16^b^	0.01

^a,b,cd^Means within the same raw arrying different letters are significantly different (p < 0.05). VM, group not included in significance (data of 1 week), but other data of 14 days.

**TABLE 5:** Effect of DF and CE with VM on relative and absolute kidney weight of the rats.

Items	Treatments					P-Value
	Control	VM	VM + DF	VM + CE	VM + DF + CE	
Absolute kidney weight (g)	1.22 ± 0.01	1.44 ± 0.13	1.28 ± 0.06	1.35 ± 0.05	1.26 ± 0.05	0.33
Relative kidney weight (g)	0.85 ± 0.04	1.03 ± 0.07	0.87 ± 0.03	0.96 ± 0.05	0.90 ± 0.04	0.14

### 3.2 Daflon and/or Centrum may mitigate vancomycin-induced alterations in hematological parameters

The impact of DF and CE co-administration with VM on hematological parameters is presented in Table 6. A significant (p ≤ 0.05) reduction in hemoglobin (Hb), red blood cell (RBC) count, and packed cell volume (PCV) was observed in the VM-treated group compared to all other groups. However, these reductions were ameliorated by the administration of DF and CE. Although the VM + DF, VM + CE, and VM + DF + CE groups showed slightly increased values compared to the control group, the differences were not statistically significant. Additionally, mean corpuscular volume (MCV), mean corpuscular hemoglobin (MCH), and mean corpuscular hemoglobin concentration (MCHC) did not differ significantly (p > 0.05) among the groups. The red cell distribution width (RDW) was slightly higher in the VM group compared to the negative control, but this difference was also not significant (p > 0.05). Co-treatment with DF and/or CE restored erythrogram indices closer to control levels and yielded marked improvements. In contrast, VM-treated rats exhibited significantly altered erythrogram parameters compared to controls. Furthermore, total white blood cell (TWBC) count, neutrophils, and the neutrophil-to-lymphocyte (N/L) ratio were significantly elevated (p ≤ 0.05) in the VM group, while lymphocyte counts were significantly reduced (p < 0.05). These changes were reversed in the VM + DF, VM + CE, and VM + DF + CE groups, which showed improved lymphocyte counts and a reduced N/L ratio compared to the VM group.

**TABLE 6:** Effect of DF and CE with VM on blood profile of male rats.

Parameters	Treatments					P-value
	Control	VM	VM + DF	VM + CE	VM + DF + CE	
RBCs (10^6^/μL)	5.67 ± 0.19^b^	5.18 ± 0. 07^c^	6.26 ± 0.13^a^	5.98 ± 0.04^ab^	5.94 ± 0.25^ab^	<0.001
Hb(g/dL)	12.80 ± 0.46^b^	10.66 ± 0.40^c^	14.04 ± 0.23^a^	12.78 ± 0.18^b^	12.56 ± 0.39^b^	<0.003
PCV (%)	36.98 ± 0. 97^a^	27.48 ± 6.03^b^	41.58 ± 0. 85^a^	39.38 ± 0.43^a^	38.48 ± 0. 89^a^	<0.020
MCV (fl)	65.14 ± 0. 80	65.74 ± 3.2	69.42 ± 0.37	65.94 ± 0. 59	69.24 ± 0.74	0.106
MCH (pg)	22.40 ± 0. 17	22.29 ± 0.61	23.40 ± 0. 55	21.22 ± 0. 26	22.42 ± 0.06	0.01
MCHC (g/dL)	34.70 ± 0. 29	29.20 ± 2.57	33.84 ± 0. 76	32.40 ± 0.22	32.70 ± 0. 53	0.02
RDW (%)	15.34 ± 0.17^ab^	15.50 ± 0.20^ab^	14.88 ± 0.22^b^	16.36 ± 0.44^a^	16.22 ± 0.47^a^	0.02
PLTs (×10^3^/μL)	969 ± 50.16^a^	956 ± 28.87^a^	891 ± 36.02^ab^	835 ± 26.47^a^	904 ± 37.48^ab^	0.04
TLC (10^3^/μL)	10.68 ± 0. 67^bc^	17.54 ± 1.44^a^	12.11 ± 0. 95^bc^	15.27 ± 1.38^ab^	17.02 ± 1.82^a^	0.05
Neutrophil (%)	7.60 ± 1.50^c^	20.00 ± 1.70^a^	10.00 ± 0. 70^bc^	11.40 ± 0.74^b^	8.60 ± 0.51^bc^	0.04
Lymphocyte (%)	86.80 ± 2.57^a^	75.80 ± 1.85^b^	85.20 ± 0.86^a^	82.20 ± 0.66^a^	85.20 ± 0.48^a^	0.01
Monocyte (%)	4.80 ± 1.06^c^	3.20 ± 0.20^a^	3.80 ± 0.20^c^	5.00 ± 0.54^b^	4.60 ± 0.40^c^	0.4
Eosinophil (%)	1.00 ± 0.00	1.00 ± 0.00	1.00 ± 0.00	1.07 ± 0.24	1.27 ± 0.24	0.004
Basophil (%)	0.00 ± 0.00	0.00 ± 0.00	0.33 ± 0.33	0.33 ± 0.33	0.33 ± 0.33	0.08
N/L ratio	0.08 ± 0.02^b^	0.27 ± 0.02^a^	0.08 ± 0.01^b^	0.08 ± 0.01^b^	0.12 ± 0.007^b^	0.02

^a,b,c^Means within the identical raw carrying dissimilar letters are significantly different (p ≤ 0.05).

### 3.3 Daflon and/or Centrum may abrogate vancomycin-induced alterations in kidney function biomarkers in rats

Significantly elevated serum levels of creatinine, uric acid, and urea (p < 0.05) in the VM-treated group indicated pronounced nephrotoxicity compared to the control rats. However, no significant differences were observed in serum albumin, globulin, or total protein levels among the treated groups. The albumin/globulin (A/G) ratio was slightly higher in most groups, though this increase was not statistically significant, except for group G5 (VM + DF + CE), which showed a significant reduction. Notably, co-administration of DF and CE with VM (VM + DF + CE) more effectively restored the kidney function markers to near-normal levels compared to VM + DF or VM + CE alone (Table 7).

**TABLE 7:** Effect of DF and/or CE on vancomycin-triggered abnormalities in serum kidney function.

Items	Treatments					P-value
	Control	VM	VM + DF	VM + CE	VM + DF + CE	
Urea (mg/dL)	39.56 ± 3.06^c^	127.00 ± 25.42^a^	77.40 ± 1.40^b^	114.00 ± 4.44^a^	68.00 ± 6.34^bc^	<0.001
Creatinine (mg/dL)	0.43 ± 0.03^c^	1.48 ± 0.20^a^	1.21 ± 0.01^a^	1.37 ± 0.04^a^	0.84 ± 0.06^b^	0.03
Uric acid (mg/dL)	5.39 ± 0.65^d^	17.20 ± 1.59^a^	8.73 ± 0.66^bc^	9.71 ± 0.21^b^	6.54 ± 0.60^cd^	<0.001
Total protein (mg/dL)	5.56 ± 0.23	5.54 ± 0.28	5.49 ± 0.24	5.58 ± 0.17	5.54 ± 0.23	0.7
Albumin (mg/dL)	4.18 ± 0.13	4.02 ± 0.16	4.06 ± 0.024	3.98 ± 0.05	3.90 ± 0.10	0.7
Globulin (mg/dL)	1.38 ± 0.07	1.52 ± 0.05	1.43 ± 0.08	1.60 ± 0.06	1.64 ± 0.06	0.05
A/G ratio	3.02 ± 0.25^ab^	2.64 ± 0.19^ab^	2.84 ± 0.16^ab^	2.50 ± 0.10^ab^	2.37 ± 0.12^b^	0.01

^a,b,c,d^Means within the same raw carrying different letters are significantly different (p ≤ 0.05).

### 3.4 Daflon and/or Centrum may ameliorate vancomycin-induced oxidative stress in the kidney by modulating oxidant and antioxidant biomarkers

VM-treated animals showed a significant increase in serum MDA levels (p ≤ 0.05), accompanied by a marked reduction in CAT and SOD activities compared to the control group. However, co-administration of DF or CE with VM reversed these alterations. Notably, group G5 (VM + DF + CE) demonstrated results that closely approached those of the normal control group and outperformed the groups receiving DF or CE alone. These findings highlight the protective role of DF and CE in mitigating VM-induced oxidative stress and preserving kidney function (Table 8).

**TABLE 8:** Effect of DF and/or CE on vancomycin -induced alterations in kidney oxidant/antioxidant biomarkers in rat.

Items	Treatments					P-Value
	Control	VM	VM + DF	VM + CE	VM + DF + CE	
MDA (nmol/g)	5.73 ± 0.53^d^	16.90 ± 0.43^a^	9.50 ± 0.25^c^	11.64 ± 0.79^b^	6.79 ± 0.29^d^	0.06
CAT (U/g)	137 ± 3.31^a^	56.10 ± 4.06^c^	98.29 ± 2.90^b^	97.23 ± 1.93^b^	138 ± 0.06^a^	<0.001
SOD (U/g)	12.90 ± 0.76^a^	3.59 ± 0.63^c^	8.53 ± 0.15^b^	8.68 ± 0.46^b^	12.70 ± 0.39^a^	0.01

^a,b,c^Means within the same raw carrying different letters are significantly different (p ≤ 0.05). VM, vancomycin; DF, daflon; CE, centrum; MDA, malondialdehyde; CAT catalase, and SOD, superoxide dismutase.

### 3.5 Gene expression

Table 9 presents the mRNA expression levels of the selected candidate genes. Compared to the control group, VM administration significantly upregulated the expression of IL-1β, TNF-α, KIM-1, RIP-1, and PARP-1 (p < 0.05). However, co-treatment with DF, CE, or their combination markedly downregulated these genes (p ≤ 0.01) relative to the VM group. Notably, group G5 (VM + DF + CE) exhibited the most favorable outcomes, with gene expression levels closely resembling those of the normal control group.

**TABLE 9:** Effect of DF and/or CE on vancomycin -induced changes in renal gene expression in rats.

Items	Treatments					P-Value
	Control	VM	VM + DF	VM + CE	VM + DF + CE	
*IL-1B/B-actin*	1.00 ± 0.00^d^	7.62 ± 0.12^a^	4.02 ± 0.02^b^	4.08 ± 0.04^b^	2.38 ± 0.02^c^	<0.001
*TNF-α/B-actin*	1.00 ± 0.00^e^	8.56 ± 0.14^a^	4.64 ± 0.24^b^	4.04 ± 0.24^c^	2.03 ± 0.26^d^	<0.001
*KIM-1/B-actin*	1.00 ± 0.00^d^	7.50 ± 0.10^a^	3.76 ± 0.024^b^	3.76 ± 0.024^b^	2.46 ± 0.024^c^	0.01
*RIP-1/B-actin*	1.00 ± 0.00^e^	7.70 ± 0.10^a^	3.94 ± 0.024^b^	3.76 ± 0.24^c^	1.86 ± 0.25^d^	<0.001
*PARP-1/B-actin*	1.00 ± 0.00^e^	9.28 ± 0.13^a^	5.08 ± 0.048^b^	4.58 ± 0.05^c^	2.26 ± 0.024^d^	0.05

^a-e^Means within the similar raw carrying unlike letters are significantly different (P ≤ 0.05).

### 3.6 Protective effects of caflon/Centrum on DNA damage assessed by comet assay

**FIGURE 1:**
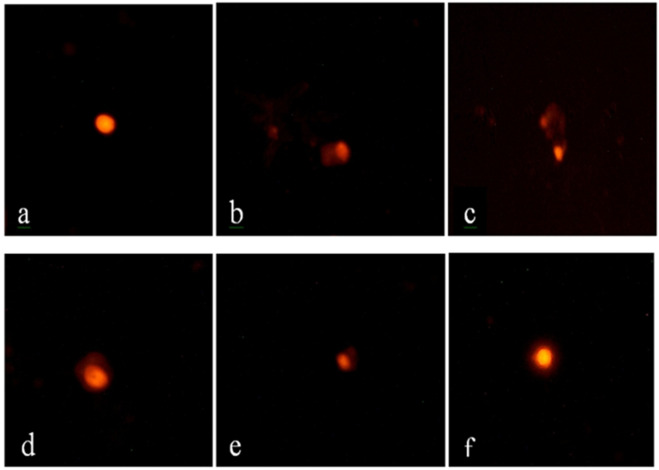
Comet assay of renal tissue from different experimental groups. **(a)** Group 1 (control) shows intact, circular nuclei with no DNA damage; **(b,c)** Group 2 (VM) displays a pronounced comet shape with extensive DNA tailing, indicating severe DNA damage; **(d)** Group 3 (VM + DF) and **(e)** Group 4 (VM + CE) exhibit moderate comet tail formation, reflecting partial DNA protection; **(f)** Group 5 (VM + DF + CE) shows mild DNA tailing, closely resembling the normal control group, suggesting a marked protective effect of the combined treatment with scale bar 50 µm.

Comet assay results are presented in Figure 1 and Table 10. Compared to the normal control group, VM-treated rats exhibited a significant increase (P ≤ 0.05) in tail length, tail DNA percentage, and tail moment, indicating a marked increase in DNA damage. Co-treatment with DF, CE, or their combination (G3, G4, and G5) significantly reduced this DNA damage. The extent of DNA damage across groups followed the order: G5 < G4 < G3 < G2. Among them, G5 (VM + DF + CE) showed the most effective DNA repair, closely approaching normal levels.

**TABLE 10:** Effect of DF and/or CE on DNA damage assessed by comet assay in rats exposed to vancomycin.

Items	Treatments					
	Control	VM	VM + DF	VM + CE	VM + DF + CE	P-Value
Tails length µm	1.12 ± 0.21^d^	15.62 ± 1.91^a^	8.35 ± 0.55^b^	10.29 ± 0.79^b^	4.82 ± 0.29^c^	<0.001
Tail DNA%	6.06 ± 1.27^e^	66.68 ± 4.49^a^	28.92 ± 1.74^c^	37.45 ± 0.69^b^	16.13 ± 1.30^d^	<0.001
Tail moment	0.09 ± 0.03^c^	5.87 ± 0.62^a^	1.28 ± 0.29^b^	1.36 ± 0.12^b^	0.54 ± 0.14^bc^	<0.001

^a-e^Means within the same raw carrying different letters are significantly different (P ≤ 0.05).

### 3.7 Histopathological assessment of vancomycin-induced kidney damage

**FIGURE 2:**
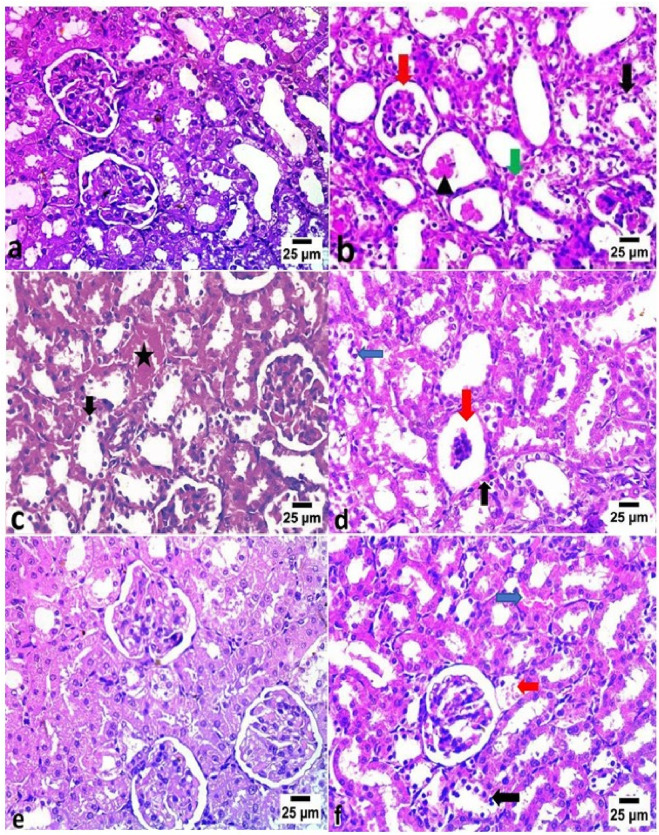
Histopathological examination of kidney tissues in different experimental groups (H&E, ×20). **(a)** The control group exhibited normal histological architecture of the glomeruli and renal tubules. **(b)** The VM group showed increased urinary space within the glomeruli (red arrow), severe vacuolar degeneration with nuclear pyknosis in the renal tubular epithelium (black arrow), renal cast formation (arrowhead), and congestion of interstitial blood vessels (green arrow). **(c)** The VM + DF group revealed congestion in peritubular blood vessels (star) and vacuolar degeneration with nuclear pyknosis in some renal tubules (black arrow). **(d)** The VM + CE group displayed glomerular atrophy (red arrow), mild peritubular blood vessel congestion (black arrow), and vacuolar degeneration with nuclear pyknosis in some renal tubules (blue arrow). **(e)** The VM + DF + CE group exhibited nearly normal histological structure of glomeruli and renal tubules. **(f)** However, mild congestion of pre-glomerular (red arrow) and peritubular blood vessels (blue arrow), along with focal vacuolar degeneration and nuclear pyknosis in some renal tubular epithelial cells (black arrow), was still observed.

Table 11 and Figure 2 illustrate the histopathological findings of kidney tissues. The control group exhibited normal renal architecture, characterized by intact renal capsules, cortex, medulla, glomeruli, tubules, and blood vessels. In contrast, VM administration caused marked pathological changes, such as widened urinary spaces within glomeruli, severe vacuolar degeneration with nuclear pyknosis in tubular epithelial cells, renal cast formation, and congestion of interstitial blood vessels. Co-treatment with DF, CE, or their combination alleviated these microscopic alterations. Notably, the VM + DF + CE group exhibited near-normal renal histology, showing superior tissue recovery compared to DF or CE treatment alone.

**TABLE 11:** Semi-quantitative scoring of renal changes in control, VM, DE, and CE-treated groups.

Items	Treatments					P-Value
	Control	VM	VM + DF	VM + CE	VM + DF + CE	
Congestion	_	***	*	**	_	0.05
Edema	_	***	*	**	_	0.005
Coagulative necrosis	_	**	*	*	*	0.001
Vacuolation	_	***	*	**	_	0.05

## 4 Discussion

Acute kidney injury (AKI) has significantly contributed to increased morbidity and mortality in recent decades. Despite advancements in critical care, patient outcomes remain suboptimal (Druml, 2014). Vancomycin is one of the most widely prescribed antibiotics, used in approximately 35% of hospitalized patients with bacterial infections due to its potent bactericidal properties and affordability (Mitevska et al., 2021). However, vancomycin-associated AKI (VM-AKI) typically manifests between 4 and 17 days after treatment initiation and may persist even after drug discontinuation, particularly in patients with underlying conditions. VM-AKI is associated with increased mortality, hospital readmissions, and prolonged stays (Kan et al., 2022). Thus, this study aimed to mitigate these adverse effects and improve renal health outcomes.

Our findings revealed that VM administration significantly elevated total white blood cell (WBC) count, neutrophils, and neutrophil/lymphocyte (N/L) ratio while markedly reducing packed cell volume (PCV), hemoglobin (Hb), red blood cells (RBCs), and lymphocytes. The observed increase in hemoglobin levels in the DF-treated group may be attributed to several potential mechanisms. Daflon, being rich in flavonoids and antioxidant compounds, can reduce oxidative stress and protect red blood cells from vancomycin-induced damage, thereby preserving their lifespan and hemoglobin content. Additionally, DF may improve microcirculation and tissue oxygenation, enhancing erythropoiesis indirectly. By mitigating renal injury and systemic oxidative stress, DF creates a more favorable environment for red blood cell production, which could explain the significant improvement in Hb levels observed in the VM + DF group (Gouda and Babiker, 2020). Unusual Drug Interaction: While there are no well-documented interactions between Daflon and Centrum that would cause a decrease in hemoglobin. However, this is not a common or established effect. Daflon is known to interact with some metabolic enzymes, so it's possible it could affect the absorption or metabolism of some components in Centrum, but a significant decrease in hemoglobin is unlikely. Lymphocytes are central humoral and cellular immunity. Uhuo et al. (2022) reported that VM at 120 mg/kg twice daily for 14 days significantly reduced hematological indices in rats, likely due to decreased erythropoietin production resulting from VM-induced AKI (Cetin et al., 2007). Our results show that co-administration of *Diosmin* (DF) and *Celvitan* (CE) effectively reversed these hematological impairments. DF, rich in flavonoids, promotes erythropoiesis and enhances iron metabolism (Olatunji et al., 2022), while also exhibiting immunomodulatory and anti-inflammatory properties. Furthermore, CE, which contains iron and multivitamins such as vitamins C and E, contributing to hematological improvement (Ekeh et al., 2019).

Biochemical analyses confirmed that VM caused significant nephrotoxicity, as evidenced by elevated serum creatinine, urea, and uric acid levels. These biomarkers are reliable indicators of impaired kidney function (Kan et al., 2022). Although total protein, albumin, and globulin levels remained relatively unchanged, hypoalbuminemia—another marker of AKI—has been strongly associated with poor outcomes (Ali et al., 2022). DF significantly reduced serum creatinine, urea, and uric acid levels, consistent with Sarheed and Al-Khalidy (2020). CE also exhibited nephroprotective effects, restoring kidney biomarkers to near-normal levels (Zeng et al., 2024). These protective effects are largely due to the antioxidant constituents of DF (mainly diosmin and hesperidin) and the vitamins and minerals in CE (Evans and Lawrenson, 2023).

Oxidative stress was further confirmed by elevated malondialdehyde (MDA) levels and reduced catalase (CAT) and superoxide dismutase (SOD) activity in VM-treated rats. Histopathological examination showed vacuolar degeneration, tubular epithelial cell pyknosis, renal cast formation, and interstitial vascular congestion. MDA, a key product of lipid peroxidation, serves as a sensitive marker for oxidative damage in the kidney (Cordiano et al., 2023). Similar findings by Qu et al. (2018) support that VM increases MDA while suppressing renal antioxidants. Mechanistically, VM increases oxidative phosphorylation in renal tubular cells, enhancing oxygen consumption and ATP production, which subsequently generates excessive reactive oxygen species (ROS), leading to DNA damage (Lee et al., 2021). Co-administration of DF and CE reversed these oxidative imbalances, enhancing CAT and SOD activities and lowering MDA levels (Sarheed and Al-Khalidy, 2020; Alahmar and Singh, 2022). The multivitamins and minerals in CE—including vitamins A, C, E, selenium, and iron—are powerful antioxidants that scavenge ROS (Uddin et al., 2021). Diosmin and hesperidin in DF also exert potent antioxidant and anti-DNA damage effects (Olatunji et al., 2022).

The VM-induced nephrotoxicity pathway involves oxidative stress and subsequent inflammation, disrupting cellular homeostasis, protein structure, and DNA integrity, and leading to apoptosis, ion imbalance, and lipid peroxidation (Lee et al., 2021). Consistent with Yu et al. (2022), we observed increased MDA and reduced SOD, GSH, and CAT in the kidneys. DF and CE ameliorated this damage, promoting DNA repair and inhibiting oxidative stress (Huwait and Mobashir, 2022; El-Gendy et al., 2024).

At the molecular level, RT-PCR revealed that VM upregulated several key genes related to apoptosis, inflammation, and kidney injury: *PARP1*, *RIPK1*, *IL-1β*, *TNF-α*, and *KIM1*. *RIPK1* is a receptor-interacting protein kinase involved in cell death and kidney injury; its suppression has been shown to improve renal outcomes (Chen et al., 2022). Our data corroborate findings by El Latif et al. (2023) linking increased *RIPK1* expression to nephrotoxicity. *PARP1*, a nuclear enzyme essential for DNA repair, becomes detrimental when excessively activated, leading to ATP depletion and cell necrosis (Song et al., 2016). Similarly, *KIM1* serves as an early and sensitive biomarker of renal tubular injury (Brilland et al., 2023), and its upregulation in our VM group aligns with results from Yu et al. (2022) and Chang et al. (2023). Proinflammatory cytokines *IL-1β* and *TNF-α* were also elevated, consistent with their role in mediating VM-induced nephrotoxicity (He et al., 2021; Yin et al., 2023; Elhemiely et al., 2025).

Treatment with DF and CE significantly downregulated these gene expressions. Their nephroprotective actions are likely due to their anti-inflammatory, anti-apoptotic, and antioxidant properties (Huwait and Mobashir, 2022; Shalkami et al., 2018; Mitra et al., 2022). The flavonoids in DF and the essential vitamins and minerals in CE played pivotal roles in mitigating oxidative DNA damage, inflammation, and apoptosis (Olatunji et al., 2022; Evans and Lawrenson, 2023).

Histopathological assessments supported the biochemical and molecular findings. Kidneys from VM-treated rats showed pronounced necrosis, congestion, degeneration, and immune cell infiltration. These outcomes are consistent with previous reports on VM-induced nephrotoxicity (Ganesh et al., 2024) and may stem from the intracellular accumulation of VM within tubular epithelial cells (Kan et al., 2022). Contrary to older hypotheses, recent data suggest that aminoglycoside toxicity is due to cytoplasmic, not lysosomal, accumulation (Randjelovic et al., 2017). DF treatment notably improved renal architecture, showing reduced tubular degeneration and preserved glomerular structure, likely due to its antioxidant and anti-inflammatory actions (Abd et al., 2023). Together, DF and CE synergistically reduced VM-induced AKI by attenuating oxidative stress, inflammation, apoptosis, and DNA damage.

The present study was conducted over a moderate experimental period; however, it would have been valuable to investigate the long-term effects of Daflon and Centrum supplementation under vancomycin-induced nephrotoxicity to determine the persistence and sustainability of their protective action. Future investigations should incorporate larger cohorts, multiple sampling intervals, molecular endpoints such as caspase-3 and cytokine profiles, and separate supplement-only groups to fully delineate mechanistic and dose-response relationships.

From a clinical perspective, Daflon and Centrum—due to their established safety and accessibility—warrant testing in controlled trials as adjunctive agents for patients undergoing treatment with nephrotoxic drugs, with emphasis on defining optimal dosing regimens and confirming translational relevance.

## 5 Conclusion

This study demonstrated that VM administration triggered apoptosis and inflammation by upregulating the gene expression of PARP1, RIP1, TNF-α, IL-1β, and KIM1. Additionally, VM-induced oxidative stress in renal tissues led to tubular degeneration and necrosis, accompanied by elevated serum levels of kidney function markers such as uric acid, urea, and creatinine. Oxidative stress markers in the kidney, including increased MDA and decreased SOD and CAT levels, further confirmed renal damage. VM also caused significant cellular DNA damage, as evidenced by the comet assay. However, treatment with DF and CE effectively alleviated VM-induced renal injury by promoting anti-inflammatory, antioxidant, and DNA repair mechanisms. Notably, the combined administration of DF and CE provided superior protective effects compared to either treatment alone.

## Data Availability

The original contributions presented in the study are included in the article/supplementary material, further inquiries can be directed to the corresponding author.

## References

[B1] Abd E.-H. Imran R. T. Jwaid A. H. (2023). The possible protective effect of daflon 500 mg on cisplatin induce nephrotoxicity in experimental rats. Res. J. Pharm. Tech. 16, 3393–3398. 10.52711/0974-360X.2023.00561

[B69] Abd El-Hafeez A. A. Khalifa H. O. Elgawish R. A. Shouman S. A. Abd El-Twab M. H. Kawamoto S. (2018). Melilotus indicus extract induces apoptosis in hepatocellular carcinoma cells via a mechanism involving mitochondria-mediated pathways. Cytotechnology 70, 831–842. 10.1007/s10616-018-0195-7 29372465 PMC5851975

[B2] Achi J. Achi N. Mallam I. (2023). Performance of growing rabbit bucks and does fed diets supplemented with brewers’ dried grains. Anim. Sci. Genet. 19, 21–29. 10.5604/01.3001.0016.2940

[B3] Al-Sroji R. Y. Al-Laham S. Almandili A. (2023). Protective effects of vitamin D3 (cholecalciferol) on vancomycin-induced oxidative nephrotoxic damage in rats. Pharm. Biol. 61, 755–766. 10.1080/13880209.2023.2204916 37139624 PMC10161947

[B4] Alahmar A. T. Singh R. (2022). Comparison of the effects of coenzyme Q10 and centrum multivitamins on semen parameters, oxidative stress markers, and sperm DNA fragmentation in infertile men with idiopathic oligoasthenospermia. Clin. Exp. Reprod. Med. 49, 49–56. 10.5653/cerm.2021.04910 35255658 PMC8923633

[B5] Alhazza I. M. Hassan I. Ebaid H. Al-Tamimi J. Alwasel S. H. (2020). Chemopreventive effect of riboflavin on the potassium bromate–induced renal toxicity *in vivo* . Naunyn Schmiedeb. Arch. Pharmacol. 393, 2355–2364. 10.1007/s00210-020-01938-7 32666286

[B6] Ali H. W. Ahmed Z. A. Aziz T. A. (2022). Effect of telmisartan and quercetin in 5-fluorouracil-induced renal toxicity in rats. J. Inflamm. Res. 15, 6113–6124. 10.2147/JIR.S389017 36386583 PMC9651059

[B7] Alkhedaide A. Elkhateeb S. A. Abass M. A. Emam M. A. Attia H. F. Amal S. (2016). Ameliorative role of grape seed extract on cadmium induced splenic toxicity in albino rats. Int. J. Innov. Res. 2, 1655–1665. Available online at: http://www.onlinejournal.

[B8] Alosaimy S. Rybakb M. J. Sakoulas G. (2024). Understanding vancomycin nephrotoxicity augmented by β-lactams: a synthesis of endosymbiosis, proximal renal tubule mitochondrial metabolism, and β-lactam chemistry. Lancet Infect. Dis. 24, e179–e188. 10.1016/S1473-3099(23)00432-2 37883984

[B52] Aswath L. Gupta S. (2009). Knowledge Management Tools and Academic Library Services London, United Kingdom: Oxford University Press. Available online at: https://www.ncbi.nlm.nih.gov/books/NBK459263/ (Accessed October 26, 2024).

[B10] Bancroft J. D. (2008). Theory and practice of histological techniques. 6th Edn. UK: Elsevier Health Sci.

[B11] Brilland B. Boud’hors C. Wacrenier S. Blanchard S. Cayon J. Blanchet O. (2023). Kidney injury molecule 1 (KIM-1): a potential biomarker of acute kidney injury and tubulointerstitial injury in patients with ANCA-glomerulonephritis. Clin. Kidney J. 16, 1521–1533. 10.1093/ckj/sfad071 37664565 PMC10468750

[B12] Caio-Silva W. da Silva Dias D. Junho C. V. C. Panico K. Neres-Santos R. S. Pelegrino M. T. (2020). Characterization of the oxidative stress in renal ischemia/reperfusion-induced cardiorenal syndrome type 3. Biomed. Res. Int. 2020, 1605358. 10.1155/2020/1605358 33102574 PMC7568802

[B14] Cetin H. Olgar Ş. Oktem F. Ciris M. Uz E. Aslan C. (2007). Novel evidence suggesting an anti-oxidant property for erythropoietin on vancomycin-induced nephrotoxicity in a rat model. Clin. Exp. Pharmacol. Physiol. 34, 1181–1185. 10.1111/j.1440-1681.2007.04695.x 17880374

[B15] Chang J. Pais G. M. Engel P. L. Klimek P. Marianski S. Valdez K. (2023). Impact of vancomycin loading doses and dose escalation on glomerular function and kidney injury biomarkers in a translational rat model. Antimicrob. Agents Chemother. 67, e0127622. 10.1128/aac.01276-22 36648224 PMC9933721

[B16] Chen L. Zhang X. Ou Y. Liu M. Yu D. Song Z. (2022). Advances in RIPK1 kinase inhibitors. Front. Pharmacol. 13, 976435. 10.3389/fphar.2022.976435 36249746 PMC9554302

[B17] Cordiano R. Gioacchino D. M. Mangifesta R. Panzera C. Gangemi S. Minciullo P. L. (2023). Malondialdehyde as a potential oxidative stress marker for allergy-oriented diseases: an update. Molecules 28, 5979. 10.3390/molecules28165979 37630231 PMC10457993

[B18] Cui H. Zhu Y. Jiang D. (2016). The RIP1–RIP3 complex mediates osteocyte necroptosis after ovariectomy in rats. PLoS One 11, e0150805. 10.1371/journal.pone.0150805 26985994 PMC4795547

[B19] Dalaklioglu S. Tekcan M. Gungor N. Celik-Ozenci C. Aksoy N. H. Baykal A. (2010). Role of the poly (ADP-ribose) polymerase activity in vancomycin-induced renal injury. Toxicol. Lett. 192, 91–96. 10.1016/j.toxlet.2009.10.002 19833176

[B20] Druml W. (2014). Systemic consequences of acute kidney injury. Curr. Opin. Crit. Care 20, 613–619. 10.1097/MCC.0000000000000150 25259720

[B21] Ekeh F. N. Ekechukwu N. E. Chukwuma C. F. Aguzie I. O. N. Ohanu C. M. Ebido C. (2019). Mixed vitamin C and zinc diet supplements co-administered with artemether drug improved haematological profile and survival of mice infected with *Plasmodium berghei* . Food Sci. Hum. Wellness 8, 275–282. 10.1016/j.fshw.2019.05.003

[B22] El Agaty S. M. (2018). Triiodothyronine attenuates the progression of renal injury in a rat model of chronic kidney disease. Can. J. Physiol. Pharmacol. 96, 603–610. 10.1139/cjpp-2017-0252 29406830

[B23] El Latif A. A. Zahra A. E. A. Badr A. Elbialy Z. I. Alghamdi A. A. A. Althobaiti N. A. (2023). The potential role of upregulated PARP-1/RIPK1 expressions in amikacin-induced oxidative damage and nephrotoxicity in wistar rats. Toxicol. Res. (Camb.) 12, 979–989. 10.1093/toxres/tfad091 37915468 PMC10615830

[B24] El-Gendy H. F. El-Bahrawy A. Mansour D. A. Sheraiba N. I. Abdel-Megeid N. S. Selim S. (2024). Unraveling the potential of *Saccharum officinarum* and *Chlorella vulgaris* towards 5-fluorouracil-induced nephrotoxicity in rats. Pharmaceuticals 17, 885. 10.3390/ph17070885 39065736 PMC11279568

[B26] Elhemiely A. A. Yahia I. Abd El Aal H. A. (2025). Carvacrol amended vancomycin -induced nephrotoxicity in rats via regulating Nrf2/HO-1, IKBβ/NF-κB and Bax/Bacl2 pathways. AIJPMS 5, 223–235. 10.21608/aijpms.2024.267591.1253

[B27] Evans J. R. Lawrenson J. G. (2023). Antioxidant vitamin and mineral supplements for slowing the progression of age-related macular degeneration. Cochrane Database Syst. Rev. 9, CD000254. 10.1002/14651858.CD000254.pub4 37702300 PMC10498493

[B28] Gan Y. Z. Azrina A. (2016). Antioxidant properties of selected varieties of lettuce (*Lactuca sativa* L.) commercially available in Malaysia. Int. Food Res. J. 23, 2357–2362. Available online at: http://www.ifrj.upm.edu.my.

[B29] Ganesh R. N. Edwards A. El Zaatari Z. Gaber L. Barrios R. Truong L. D. (2024). Vancomycin nephrotoxicity: a comprehensive clinico-pathological study. PLoS One 19, e0295136. 10.1371/journal.pone.0295136 38452051 PMC10919848

[B30] Góth L. (1991). A simple method for determination of serum catalase activity and revision of reference range. Clin. Chim. Acta 196, 143–151. 10.1016/0009-8981(91)90067-m 2029780

[B31] Gouda E. Babiker F. (2020). Micronized flavonoid fraction daflon 500 protects heart againstischemia–reperfusion injury: an old medicine for a new target. All Life 13, 556–568. 10.1080/26895293.2020.1832921

[B32] He J. Xu W. Zheng X. Zhao B. Yu P. Deng S. (2021). Vitamin C reduces vancomycin-related nephrotoxicity through the inhibition of oxidative stress, apoptosis, and inflammation in mice. Ann. Transl. Med. 9, 1319–11. 10.21037/atm-21-3294 34532456 PMC8422136

[B33] Humanes B. Jado J. Camaño S. López-Parra V. Torres A. M. Álvarez-Sala L. A. (2015). Protective effects of cilastatin against vancomycin-induced nephrotoxicity. Biomed. Res. Int. 2015, 704382. 10.1155/2015/704382 26504822 PMC4609390

[B34] Huwait E. Mobashir M. (2022). Potential and therapeutic roles of diosmin in human diseases. Biomedicines 10, 1076. 10.3390/biomedicines10051076 35625813 PMC9138579

[B35] Jackson P. Pedersen L. M. Kyjovska Z. O. Jacobsen N. R. Saber A. T. Hougaard K. S. (2013). Validation of freezing tissues and cells for analysis of DNA strand break levels by comet assay. Mutagenesis 28, 699–707. 10.1093/mutage/get049 24136994 PMC3804896

[B36] Juszczak A. B. Kupczak M. Konecki T. (2023). Does vitamin supplementation play a role in chronic kidney disease? Nutrients 15, 2847. 10.3390/nu15132847 37447174 PMC10343669

[B37] Kan W. C. Chen Y. C. Wu V. C. Shiao C. C. (2022). Vancomycin-associated acute kidney injury: a narrative review from pathophysiology to clinical application. Int. J. Mol. Sci. 23, 2052. 10.3390/ijms23042052 35216167 PMC8877514

[B38] Kartavenka K. Panuwet P. Yakimavets V. Jaikang C. Thipubon K. D'Souza P. E. (2020). LC-MS quantification of malondialdehyde-dansylhydrazine derivatives in urine and serum samples. J. Anal. Toxicol. 44, 470–481. 10.1093/jat/bkz112 31897465 PMC8269965

[B39] Kayoumu M. Iqbal A. Muhammad N. Li X. Li L. Wang X. (2023). Phosphorus availability affects the photosynthesis and antioxidant system of contrasting low-P-tolerant cotton genotypes. Antioxidants 12, 466. 10.3390/antiox12020466 36830024 PMC9952849

[B40] Kucukler S. Darendelioğlu E. Caglayan C. Ayna A. Yıldırım S. Kandemir F. M. (2020). Zingerone attenuates vancomycin-induced hepatotoxicity in rats through regulation of oxidative stress, inflammation and apoptosis. Life Sci. 15, 118382. 10.1016/j.lfs.2020.118382 32898532

[B41] Lee H. S. Kim S. M. Jang J. H. Park H. D. Lee S. Y. (2021). Serum 5-hydroxyindoleacetic acid and ratio of 5-hydroxyindoleacetic acid to serotonin as metabolomics indicators for acute oxidative stress and inflammation in vancomycin-associated acute kidney injury. Antioxidants 10, 895. 10.3390/antiox10060895 34199555 PMC8228749

[B42] Li G. Walker M. J. De Oliveira D. M. P. (2022). Vancomycin resistance in enterococcus and *Staphylococcus aureus* . Microorganisms 11, 24. 10.3390/microorganisms11010024 36677316 PMC9866002

[B43] Li X. H. Luo Y. Z. Mo M. Q. Gao T. Y. Yang Z. H. Pan L. (2024). Vitamin D deficiency may increase the risk of acute kidney injury in patients with diabetes and predict a poorer outcome in patients with acute kidney injury. BMC Nephrol. 25, 333. 10.1186/s12882-024-03781-x 39375595 PMC11460229

[B44] Liu M. Dudley S. C. (2020). Magnesium, oxidative stress, inflammation, and cardiovascular disease. Antioxidants 9, 907. 10.3390/antiox9100907 32977544 PMC7598282

[B45] Livak K. J. Schmittgen T. D. (2001). Analysis of relative gene expression data using real-time quantitative PCR and the 2(-Delta Delta C(T)) method. Methods 25, 402–408. 10.1006/meth.2001.1262 11846609

[B46] Misra H. P. Fridovich I. (1972). The role of superoxide anion in the autoxidation of epinephrine and a simple assay for superoxide dismutase. J. Biol. Chem. 247, 3170–3175. 10.1016/s0021-9258(19)45228-9 4623845

[B47] Mitevska E. Wong B. Surewaard B. G. J. Jenne C. N. (2021). The prevalence, risk, and management of methicillin-resistant *Staphylococcus aureus* infection in diverse populations across Canada: a systematic review. Pathogens 10, 393. 10.3390/pathogens10040393 33805913 PMC8064373

[B48] Mitra S. Paul S. Roy S. Sutradhar H. Bin Emran T. Nainu F. (2022). Exploring the immune-boosting functions of vitamins and minerals as nutritional food bioactive compounds: a comprehensive review. Molecules 27, 555. 10.3390/molecules27020555 35056870 PMC8779769

[B49] Mohamed N. A. Hassan M. H. Saleem T. H. Mohamed S. A. El-Zeftawy M. Ahmed E. A. (2021). KIM-1 and GADDI-153 gene expression in paracetamol-induced acute kidney injury: effects of N-acetylcysteine, N-acetylmethionine, and N-acetylglucosamine. Turk. J. Biochem. 47, 409–416. 10.1515/tjb-2021-0233

[B50] Olatunji A. O. Alegbe R. O. Ambali S. F. Shittu M. Akorede G. J. Aremu A. (2022). Co-administration of *Hibiscus sabdariffa* linn and Daflon-500® mitigates weight loss, hyperglycemia, hematological and oxidative changes in male rats with type-2 diabetes. Phytomedicine Plus 5, 100699. 10.1016/j.phyplu.2024.100699

[B51] Paget G. E. Barnes I. M. (1964). Interspecies dosage conversion scheme in evaluation of results and quantitative application in different species in (evaluation of activities:Pharmacometrics), 1. London and New York: Academic Press, 160–163.

[B70] Patel S. Preuss C. V. Bernice F. (2024). Vancomycin, Treasure Island, FL: StatPearls Publishing, Available online at: https://www.ncbi.nlm.nih.gov/books/NBK459263/ . 29083794

[B53] Qu S. Dai C. Lang F. Hu L. Tang Q. Wang H. (2018). Rutin attenuates vancomycin-induced nephrotoxicity by ameliorating oxidative stress, apoptosis, and inflammation in rats. Antimicrob. Agents Chemother. 63, e01545-18. 10.1128/AAC.01545-18 30397060 PMC6325182

[B54] Randjelovic P. Veljkovic S. Stojiljkovic N. Sokolovic D. Ilic I. (2017). Gentamicin nephrotoxicity in animals: current knowledge and future perspectives. EXCLI J. 16, 388–399. 10.17179/excli2017-165 28507482 PMC5427480

[B55] Sarheed N. M. Al-Khalidy S. A. (2020). The protective effect of daflon against the renal-hepatic toxicity induced by methotrexate in rabbits. Pak. J. Biotechnol. 17, 17–23. 10.34016/pjbt.2020.17.1.3

[B56] Shalkami A. Hassan M. Bakr A. (2018). Anti-inflammatory, antioxidant and anti-apoptotic activity of diosmin in acetic acid-induced ulcerative colitis. Hum. Exp. Toxicol. 37, 78–86. 10.1177/0960327117694075 29187079

[B57] Singh N. P. McCoy M. T. Tice R. R. Schneider E. L. (1988). A simple technique for quantitation of low levels of DNA damage in individual cells. Exp. Cell Res. 175, 184–191. 10.1016/0014-4827(88)90265-0 3345800

[B58] Soltani R. Khorvash F. Meidani M. Badri S. Alaei S. Taheri S. (2020). Vitamin E in the prevention of vancomycin-induced nephrotoxicity. Res. Pharm. Sci. 15, 137–143. 10.4103/1735-5362.283813 32582353 PMC7306246

[B59] Song H. Yoon S. P. Kim J. (2016). Poly(ADP-ribose) polymerase regulates glycolytic activity in kidney proximal tubule epithelial cells. Anat. Cell Biol. 49, 79–87. 10.5115/acb.2016.49.2.79 27382509 PMC4927434

[B60] Toprak T. Sekerci C. A. Aydın H. R. Ramazanoglu M. A. Arslan F. D. Basok B. I. (2020). Protective effect of chlorogenic acid on renal ischemia/reperfusion injury in rats. Arch. Ital. Urol. 92 (2). 10.4081/aiua.2020.2.153 32597123

[B61] Uddin S. M. N. Sultana F. Uddin M. G. Dewan S. M. R. Hossain M. K. Islam M. S. (2021). Effect of antioxidant, malondialdehyde, macro-mineral, and trace element serum concentrations in Bangladeshi patients with schizophrenia: a case-control study. Health Sci. Rep. 4, e291. 10.1002/hsr2.291 34013069 PMC8112814

[B62] Uhuo E. N. Egba S. I. Nwuke P. N. Odinamadu H. (2022). Renoprotective effects of *Adansonia digitata* leaf extract on renal function and histopathological changes in vancomycin-induced nephrotoxicity in Wistar rats. Comp. Clin. Pathol. 31, 229–242. 10.1007/s00580-022-03325-5

[B63] Van Groesen E. Innocenti P. Martin N. I. (2022). Recent advances in the development of semisynthetic glycopeptide antibiotics: 2014–2022. ACS Infect. Dis. 8, 1381–1407. 10.1021/acsinfecdis.2c00253 35895325 PMC9379927

[B64] Wang F. Zhou H. Olademehin O. P. Kim S. J. Tao P. (2018). Insights into key interactions between vancomycin and bacterial cell wall structures. ACS Omega 3, 37–45. 10.1021/acsomega.7b01483 29399648 PMC5793038

[B65] Yin J. Wang C. Vogel U. Ma Y. Zhang Y. Wang H. (2023). Common variants of pro-inflammatory gene IL1B and interactions with PPP1R13L and POLR1G in relation to lung cancer among northeast Chinese. Sci. Rep. 13, 7352. 10.1038/s41598-023-34069-z 37147350 PMC10161999

[B66] Yu P. Luo J. Song H. Qian T. He X. Fang J. (2022). N-acetylcysteine ameliorates vancomycin-induced nephrotoxicity by inhibiting oxidative stress and apoptosis in the *in vivo* and *in vitro* models. Int. J. Med. Sci. 19, 740–752. 10.7150/ijms.69807 35582415 PMC9108398

[B67] Zeng H. Liu Z. He Y. Chen H. He J. Liu M. (2024). Multivitamins co-intake can reduce the prevalence of kidney stones: a large-scale cross-sectional study. Int. Urol. Nephrol. 56, 2991–3001. 10.1007/s11255-024-04021-9 38564076

[B68] Zheng J. Yang X. Zhang C. Zhang W. Hu Y. Zeng L. (2024). Icariin reduces cadmium-induced renal injury in rats. Food Chem. Toxicol. 193, 114964. 10.1016/j.fct.2024.114964 39197519

